# Editorial: Bronchopulmonary dysplasia: latest advances

**DOI:** 10.3389/fped.2023.1303761

**Published:** 2023-11-13

**Authors:** Shahana Perveen, Chung-Ming Chen, Hisanori Sobajima, Xiaoguang Zhou, Jia-Yuh Chen

**Affiliations:** ^1^ Department of Pediatrics, Cohen Children Medical Center, Manhasset, NY, United States; ^2^ Department of Pediatrics, Zucker School of Medicine at Hofstra/Northwell, Manhasset, NY, United States; ^3^ Department of Pediatrics, Taipei Medical University, Taipei, Taiwan; ^4^ Division of Neonatology, Department of Pediatrics, Saitama Medical Center, Saitama Medical University, Saitama, Japan; ^5^ Department of Neonatology, The Eighth Affiliated Hospital, Sun Yat-Sen University, Kangdon, China; ^6^ Division of Neonatology, Changhua Christian Children’s Hospital, Changhua, Taiwan

**Keywords:** bronchopulmonary dysplasia, mechanical ventilation, oxygen therapy, prematurity, lung injury

**Editorial on the Research Topic** Bronchopulmonary dysplasia: latest advances

Bronchopulmonary dysplasia (BPD) is a complex and devastating condition related to prematurity. BPD is the most common chronic condition of infancy and is associated with significant morbidity and mortality and high financial burden on healthcare systems, families, and communities (1). Infants are not born with BPD; the condition evolves with time with prolonged use of mechanical ventilation and oxygen therapy leading to lung damage. The pathogenesis of BPD is complex, poorly understood, and multifactorial. The incidence has not changed in last two decades despite many advances in neonatology because of the improved survival of extreme preterm infants, including babies less than 500 g (2). Advances in technologies and improving clinical care have led to a lower incidence of “Classic BPD” described as heterogeneity and severe airway epithelial lesions, airway smooth muscle hyperplasia, extensive alveolar septal fibrosis, and hypertensive remodeling of the pulmonary arteries (3). It has been replaced it with a “New BPD” described as less heterogeneity, with large simplified alveolar structures, reduced and dysmorphic vascular bed and mild airway smooth muscle thickening (4) which present with distinct clinical and pathological features in surviving infants (5). Regardless of changing definition BPD is a chronic condition which mainly affects premature infants with disparity in lung injury and repair processes in the presence of immature developing lung (6). The overall incidence of BPD in infants born prematurely <28-week gestation age is approximately 48%–68% and inversely related to gestational age (7). The pathogenesis of BPD remains unclear and involves several prenatal as well as post-natal factor in the presence of prematurity leading to lung damage (8). These multiple factors, including mechanical ventilation, oxygen therapy, infections, exposure to toxins and genetic factors play a key role in the development of BPD. The diagnosis of BPD in preterm infants remains associated with significant short term and long-term morbidity (2). The general approach is to prevent lung injury with optimal ventilator adjustment, prevent infection and promoting lung growth by providing adequate nutrition.

The research topic “Bronchopulmonary Dysplasia: Latest Advances” presents a total of 12 articles focusing on underlying etiology, pathogenesis, prevention bundles for improving the understanding and management strategies of the BPD. One of the articles “Association of newer definitions of bronchopulmonary dysplasia with pulmonary hypertension and long-term outcomes” by Hwang et al. describes the importance of using new evolving definition of BPD and its impact on predicting long term outcome and development of pulmonary hypertension. This retrospective study enrolled preterm infants born at <32-week GA B/W 2014–2018 at Guri Hospital, Korea. This is the first study that compares the different criteria of BPD regarding pulmonary hypertension in preterm infants and showed an association between recently described criteria for defining BPD as NICHD 2018 & NICHD 2019 criteria with severity of BPD and later outcome including pulmonary hypertension in preterm infants. This is an important concept to recognize as BPD is now described as a form of pulmonary vascular disease (9) and development of pulmonary hypertension could be spectrum of vascular disease with increasing severity and poor prognosis (10).

“A prediction nomogram for moderate-to-severe bronchopulmonary dysplasia in preterm infants <32 weeks of gestation: A multicenter retrospective study” by Zhang et al. presents a multicenter retrospective study conducted with aim to develop a dynamic nomogram for early prediction of BPD using perinatal factors in preterm infants born at <32 weeks' gestation. This was conducted at three hospitals in China between January 2017 and December 2021. There are several prediction models in extreme premature babies predicting the BPD and poor outcome (11, 12), however metanalysis have showed the limited utility and validity in these prediction models (13, 14). Using machine learning the authors indentified GA, Apgar 5-min score, early onset sepsis score (EOS), small for gestational age (SGA), and duration of invasive ventilation as predictors for BPD. Predicting significant BPD as early as within 7 days of life is an important aspect in future management of the babies at risk for BPD.

“Association between the development of bronchopulmonary dysplasia and platelet transfusion: a protocol for a systematic review and meta-analysis” by Chioma et al. describes an potentially important link to development of bronchopulmonary dysplasia. Platelets play a role in the formation of pulmonary blood vessels and thrombocytopenia has been described with pulmonary disease including BPD (15). Chioma et al. describe a protocol and plan to evaluate the correlation of platelet transfusion in preterm infant with development of BPD based on a systemic review and metanalysis. As platelet transfusion associated with release of bioactive factors can enhances oxidative stress leading to altered angiogenesis and BPD.

“Development of a novel humanized mouse model to study bronchopulmonary dysplasia” by Birkett et al. addresses the role of fetal circulating monocytes from cord blood in the pathogenesis of bronchopulmonary dysplasia. It also explores the relationship of chorioamnionitis and pre-eclampsia on fetal circulating monocytes using humanized mouse model. The study describes that fetal monocyte exposed to preeclampsia shows accelerated alveolarization as compared to exposure to chorioamnionitis which causes arrest of alveolarization. This study provides a remarkably interesting and novel approach to study basic underlying molecular mechanism and lung disease. Fetal monocytes play a role in in early lung development in humanized mouse model of BPD. Fetal monocyte induced vascular development was inhibited by hyperoxia and provide a platform for further exploration and patient targeted therapeutic approach and interventions.

“Two-stage learning-based prediction of bronchopulmonary dysplasia in very low birth weight infants: a nationwide cohort study” by Hwang et al. This study describes the development of machine-based learning model for the prediction of bronchopulmonary dysplasia and its severity through two stage approach incorporating the duration of respiratory support and prenatal and early postnatal variables. This study provides potential useful tool for clinician for early prediction of BPD and its severity with high predictive accuracy. There is great need for a reliable prediction model for BPD in preterm infants and future studies evaluating this model may be very helpful in achieving and validating this prediction model in neonatal intensive care units.

Extracellular vesicles (EVs) are a diverse array of nano-sized membranous structures that are becoming recognized as intercellular and inter-organ communication mediators. Wu et al. describe the isolation methods, characterization techniques, and the functions of EVs. The phospholipid membrane of EVs protects their payload from the extracellular environment, allowing for safe transit and distribution of their cargo to local or distant target cells and consequently, alters the target cell's gene expression, signaling pathways, and function. The extremely selective, sophisticated network via which EVs promote cell communication and modify cellular processes makes EVs as pathogenic messengers, biomarkers, and potential treatments for newborn lung disorders.

Various biomarkers have been studied for early prediction of BPD (9, 16–18). Biomarkers may be valuable for early diagnosis of BPD, enabling initiation of therapies to reduce the incidence of BPD and long-term cardiorespiratory impairment. Wang et al. describe that a combining clinical data, molecular biomarkers, and echocardiogram measurements can be valuable in predicting BPD. The tricuspid regurgitation jet, N-terminal-pro-B-brain natriuretic peptide (NT-pro BNP), ventilator associated pneumonia, days of FiO_2_ ≧40%, red blood cell volume, and proportion of infants who receive total enteral milk (120 Kcal/kg/day) ≧24 days after birth are the most practical factors for predicting the risk of BPD. Gaertner et al. use electrical impendence tomography (EIT) to measure regional ventilation distribution and overall lung aeration. They describe that EIT markers of aeration at 30 min after birth accurately predict the need of oxygen supplement at 28 days of age but not the need of intubation or BPD.

## Conclusion

The Research Topic “Bronchopulmonary dysplasia: Latest Advances” goal was to incorporate the most recent advances in basic science research, translational research, clinical data, and preventive measures towards the better understanding and management of patients with BPD. Even though there have been promising advancements in understanding and management of the BPD, this condition remains complex and without any definitive cure. Continued research and collaboration among healthcare professionals and researchers are crucial to further improve the understanding, prevention, and treatment of bronchopulmonary dysplasia.
